# Identification of UAP1L1 as a critical factor for prostate cancer and underlying molecular mechanism in tumorigenicity

**DOI:** 10.1186/s12967-022-03291-0

**Published:** 2022-02-15

**Authors:** Xing-cheng Wu, Yu-zhong Yu, Yu-zhi Zuo, Xian-Lu Song, Zhi-en Zhou, Yu Xiao, Dao-sheng Luo, Wei-gang Yan, Shan-Chao Zhao

**Affiliations:** 1 Department of Urology, Peking Union Medical College Hospital, Chinese Academy of Medical Sciences, Beijing, 100720 China; 2 Department of Urology, Nanfang Hospital, Southern Medical University, Guangzhou, 510515 China; 3 Department of Radiotherapy, Affiliated Cancer Hospital & Institute of Guangzhou Medical University, Guangzhou, China; 4 Department of Pathology, Peking Union Medical College Hospital, Chinese Academy of Medical Sciences, Beijing, China; 5 Department of Urology, Dongguan Hospital, Southern Medical University, Guangzhou, China

**Keywords:** Prostate cancer, UAP1L1, CDCA8

## Abstract

**Background:**

Prostate cancer is the second most common cancer in men, and some new target genes are needed to predict the risk of prostate cancer progression and the treatment.

**Methods:**

In this study, the effects of UAP1L1 (UAP1-like-1) on prostate cancer were investigated by detecting the proliferation, migration, invasion and apoptosis of prostate cancer cells in vitro using MTT, wound healing, Transwell and flow cytometry assay, and the tumor growth in vivo. The downstream genes and pathways of UAP1L1 were explored using Ingenuity Pathway Analysis (IPA), and screened by qRT-PCR and western blot. The effects of CDCA8 on prostate cancer cells were also verified in vitro, which was through detecting the change of proliferation, migration, invasion and apoptosis of prostate cancer cells after CDCA8 knockdown.

**Results:**

The results indicated that UAP1L1 promoted the proliferation, migration and invasion of prostate cancer cells, which was inhibited by downregulating CDCA8. Furthermore, the promotion of CDCA8 knockdown on cell apoptosis was reduced when UAP1L1 was simultaneously overexpressed.

**Conclusions:**

In conclusion, the results in this study revealed that UAP1L1 promoted the progression of prostate cancer through the downstream gene CDCA8.

**Supplementary Information:**

The online version contains supplementary material available at 10.1186/s12967-022-03291-0.

## Background

Glycosylation is the most abundant and diverse post-translational modification of proteins, and reflects the coordinated effort of a complex array of enzymes, organelles, and other factors that are essential to successfully generate carbohydrate-associated post-translational modification [1]. The changes of cellular glycosylation have recently received attention as a key component of cancer progression. Alterations in glycosylation not only directly impact cell growth and survival but also facilitate tumor-induced immunomodulation and eventual metastasis. For instance, inhibition of complex *O*-glycan formation results in impaired sensitivity of DR4 and DR5, apoptosis-inducing receptors that relay signaling by TRAIL which normal induces death in cancer cells [2, 3]. Many of these changes may contribute to cancer progression, and may also serve as a distinct feature of cancers and therefore provide novel diagnostic and therapeutic targets. For example, recent study indicate that the Tn and STn carbohydrate antigens may be the most commonly altered *O*-glycansongly on glycoproteins and significantly up-regulated in many types of tumors, such as colon, breast and lung [4, 5]. Therefore, Tn and STn may serve as a prognostic marker and therapeutic target.

UAP1-like-1 (UAP1L1) is a protein that shares ~ 59% sequence identity with UDP-*N*-acetylglucosamine pryophosphorylase-1 (UAP1), which is directly involved in the synthesis of the sugar donor (UDP-GlcNac) for *N*-acetylglucosamine modification (*O*-GlcNAclation) of proteins in the cytoplasm, nucleus, and mitochondria, as well as for *O*- and *N*-linked protein glycosylation in the endoplasmic reticulum and the Golgi apparatus [1, 6, 7]. *O*-GlcNAc transferase (OGT) is the single enzyme that conducts the transfer function of GlcNAc from UDP-GlcNAc to serines and threonines of many protein substrates [8]. UDP-GlcNAc is synthesized by an enzyme named UAP1 or GlcNac1P uridyltransferase, depending on the direction of the reaction considered [9]. UAP1L1 has been reported to correlate with breast tumor patient relapse-free survival, and directly interacts with OGT to mediated hepatocellular carcinoma cell proliferation [10, 11].

However, the association of UAP1L1 with prostate cancer and functions remain unclear. Here we reported the study of UAP1L1 in prostate cancer and provide evidence that UAP1L1 has a critical role in tumorigenesis through targeting CDCA8, which is essential for the growth of at least two prostate cancer cell lines.

## Methods

### Cell culture

The prostate cancer cell lines DU145, PC-3, and C4-2 were purchased from BeNa Culture Collection (Beijing, China), and all of them were maintained in the RPMI-1640 (HyCline Logan, UT, USA) with 10% fetal calf serum (FBS). Cells were cultured in an incubator containing 5% CO_2_ at 37 °C.

### Immunohistochemical staining

Paraffin-embedded human prostate cancer tissue chip was purchased from Xi’an Alena Biotechnology Co. LTD (BNS19011, PR1921b). There were a total of 238 samples in this study, among which 158 were prostate cancer tissues and other 80 were normal prostate tissues. All of the patients were signed the informed consent and the clinicopathologic characteristic of these patient was collected for statistical analysis. This study was approved by the Ethics committee of Peking Union Medical College Hospital.

The paraffin-embedded tissues sections of human prostate cancer tissues and normal prostate tissues were dewaxed by xylene 10 min per time, antigen retrieval was carried out by citric acid buffer for 20 min at 120 °C. The sections were blocked by 3% H_2_O_2_ for 5 min, incubated with primary antibodies overnight at 4 °C, and then incubated with second antibodies for 1 h at 37 °C. DAB was used to stain sections for 5 min in the dark, and hematoxylin was used for counterstaining. After washing, sections were dehydrated by alcohol, sealing with neutral gum, and observed via microscopic. Specimens were classified according to the sum of the staining intensity and staining extent scores. The details information of primary antibodies and second antibodies showed in Additional file 1: Table S1.

### Lentiviral plasmid construction and infection

Using UAP1L1 and CDCA8 genes as templates, the RNA interference target sequences were designed, which were provided in Additional file 1: Table S2. Recombinant lentivirus vector was obtained by cloning the overexpression sequence and shRNA into the pGCSIL-green fluorescent protein lentivirus vector with Agel/EcoRI site. Recombinant lentiviral vector and packaging vectors were co-infected into 293 T cells when cell density reached 70%. The cells were cultured for 48 h in an incubator with 5% CO_2_ at 37 °C. Then lentivirus particles were collected and purified by ultracentrifugation. The titer of lentivirus was measured using end-point dilution assay. Prostate cancer cells were placed into 6-well plates, and infected with lentivirus. 72 h after infection, cells were observed under a fluorescence microscope (MicroPublisher 3.3RTV; Olympus, Tokyo, Japan), and the infection efficiency was assessed.

### qRT-PCR

Cells were collected and centrifugated to extract total RNA with TRlzol^®^ reagent (Thermo Fisher Scientific). Nanodrop 2000/2000C spectrophotometry (Thermo Fisher Scientific) served to analyse the concentration and purity of the RNA. 2 μg total RNA was used for reverse transcription into cDNA by using the Promega M-MLV Reverse Transcriptase Kit in accordance with the manufacturer’s instruction. Real-PCR was carried out using SYBP Premix Ex Taq TMII. GAPDH was served as internal parameter, and 2^−∆∆Ct^ was performed to calculate the relative expression level of each gene. The primer sequences used were listed in Additional file 1: Table S3.

### Western blot

Cells after lentivirus infection were lysed by 1× Lysis Buffer (Cell Signal Technology, Danvers, MA) and the concentration of total protein was measured by BCA Protein Assay Kit (HyClone-Pierce, Logan, UT, USA). 20 μg proteins were separated by 10% sodium dodecyl sulfate-polyacrylamide gel electrophoresis (SDS-PAGE), and transferred to the ployvinylidene fluoride (PVDF) membrane. The membranes were blocked by TBST with 5% skim milk overnight at 4 °C, incubated with primary antibodies for 2 at room temperature, and then incubated with second antibodies for 2 at room temperature. ECL-Plus™ Western blotting system (GE Healthcare Life Sciences, USA) was used for membranes color rendering, and proteins were detected with an X-ray imaging analyzer (Kodak). All of the information about primary antibodies and second antibodies was provided in Additional file 1: Table S1.

### MTT assay

Cells in the logarithmic growth phase were seeded into 96-well plates at a concentration of 2000 cells/well, and each group had 3 replicate wells. 4 h before the termination of culture, 20 μL 5 mg/mL MTT (Genview) was added into each well. Then MTT and medium were completely discarded and 100 μL dimethyl sulfoxide (DMSO, Sigma-Aldrich, St. Louis, MO, USA) was added. The absorbance at 490 nm was measured by using microplate reader (Tecan) when cells were cultured for 1, 2, 3, 4, and 5 days.

### Colony formation assay

3 days after infection with lentivirus with UAP1L1 RNAi or control sequence, DU 145 or PC-3 cells were inoculated in 6-well plates with 1000 cells per well, and cultured for another 8 days, during which the medium was changed every 3 days. 1 mL 4% paraformaldehyde was used to fix cells for 30–60 min, and then 500 μL Giemsa was employed to stain cells for 10–20 min. After washing by ddH_2_O, cells were taken pictures with a digital camera for counting the number of cell colony. The clone formation standard contains were that the number of cells was more than 50.

### Transwell assay

100 μL mixture of Matrigel (BD Biosciences, San Jose, CA, USA) and serum-free medium (the dilution ratio was 1:6) was added into the upper chamber, and incubated for 4–6 h in an incubator at 37 °C until the gel formation. 100 μL serum-free medium was then added to rehydrate the gelled matrigel. 600 μL medium with 30% FBS was added into the lower chamber, while 100 μL cell suspension (1 × 10^5^ cells) was added into the upper chamber for 24 h. After removing cells that did not penetrate the gelled matrigel using a cotton swab, cells adhering to the gelled matrigel were fixed by 4% paraformaldehyde and stained by 0.1% crystal violet for 20 min. Finally, five field of view was randomly selected and photographed with a microscope. The invading cells were counted.

### Wound healing assay

After digestion cells in logarithmic growth with trypsin, 5 × 10^4^ cells were counted and placed in 96-well plates. When the cells reached more than 90% confluence, 96 Wounding Replicator (VP scientific, Inc., San Diego, California, USA) was used to align the central part of the lower end of 96-well plate, gently push upward to form a line wound on the cell layer. The plates were washed by serum-free medium. After that, cells were cultured in an incubator with 5% CO_2_ at 37 °C. At the time point (0 h, 24 h, 48 h), fluorescence microscopy was used to take photographs. Cellomics (Thermo Fisher Scientific, Waltham, MA, USA) was used to analyze cell migration area and calculate the cell migration rate.

### Flow cytometry for cell apoptosis

Prostate cancer cells were placed into 6-well plates for 5 days, when the coverage rate of cells reached 85%. 1× binding buffer washed the cells once after D-Hanks, which was pre-cooled at 4 °C, washed the cells, and then cells were centrifuged for 3 min at 1300 rpm. 200 μL 1× binding buffer was used to resuspend cells, and 10 μL Annexin V-APC (eBioscience, California, USA) was added staining for 10–15 min at room temperature in the dark. Flow cytometry (Millipore, Schwalbach, Germany) was employed to detect and evaluate cell apoptosis rate. Each experiment was repeated 3 times.

### Human Apoptosis Antibody Array analysis

Human Apoptosis Antibody Array (ab134001, Abcam, Cambridge, MA, USA) was used to preliminarily investigate the apoptosis mechanism regulating by UAP1L1 in PC-3 cells. After infection with shCtrl or shUAP1L1, PC-3 cells were lysed and the total proteins were extracted from cell lysates. Each membrane was blocked by 2 mL 1× blocking buffer for 30 min at room temperature, incubated with 1.2 mL sample overnight at 4 °C, and then washed by Wash Buffer I and Wash Buffer II. 1 mL 1× Biotin-conjugated Anti-Cytokines was added to every membrane and incubated overnight at 4 °C. After washing off the Biotin-conjugated Anti-Cytokines, the membranes were incubated with 1.5 mL 1× Streptavidin-HRP for 2 h at room temperature. Finally, the signals were detected using the chemiluminescence imaging system.

### Ingenuity Pathway Analysis (IPA)

Total RNA was extracted from UAP1P1 knockdown PC-3 cells according to the manufacturer’s guidelines, which were used for RNA sequencing. The gene clustering analysis and differential expression assessment were performed by Limma package of R studio with the screening criteria: *P* < 0.05 and |log_2_Fold Change|≥ 2. Ingenuity Pathway Analysis (IPA) was used to analyze differentially expressed genes (DEGs) in UAP1L1 knockdown cells and the results showed the classical pathways, disease and function, and the interaction between DEGs and pathways.

### Co-immunoprecipitation

C4-2 cells were lysed with IP lysis and the protein concentration was determined by the BCA Protein Assay Kit (HyClone-Pierce, Logan, UT, USA). 1.0 mg of protein and the antibody were incubated overnight with rotation at 4 ℃. The protein lysate was incubated with 20 μL beads at 4 ℃ for 2 h. Appropriate amount of IP lysis was added, mixed well, centrifuged and discarded the supernatant. After that, the samples were subjected to SDS-PAGE electrophoresis and transferred to PVDF membrane. After the whole membrane was sealed with 5% skim milk at room temperature for 1 h, the membrane was incubated with primary antibody at 4 ℃ overnight. 1× TBST for 3 times, 5 min each time. The second antibody was incubated at room temperature for 2 h and washed with 1× TBST for 3 times, 5 min each time. At last, immunocoloration was performed and photos were taken.

### Nude mice xenograft model

20 BALB/c nude mice (4-week old) were purchased from Shanghai SLAC Laboratory Animal Co., Ltd (Shanghai, China), and separated into two group (shCtrl and shUAP1L1) randomly. 5 × 10^6^ PC-3 cells, resuspended by D-Hanks, were injected subcutaneously into the armpit of the right forelimb of mice. The mice were fed for 35 days, and data were collection from day 13 after injection. The mice were imaged in vivo before sacrifice. 10 min before in vivo imaging, mice were anesthetized by intraperitoneal injection of 0.7% pentobarbital sodium. Tumor burden was assessed by bioluminescence imaging and analyzed by IVIS Spectrum Imaging System (emission wavelength, 510 nm). The mice were sacrificed 35 days after injection by intrapertoneal injection of excessive pentobarbital sodium (120 mg/kg), and then tumors were removed for photographing and weighting. Besides, the length and width of tumors were measured by a caliper at 13, 19, 25, 29, 33, 35 days after injection, and the volume was calculated by the equation π/6 × L × W^2^ (L represent longest dimension; W means dimension perpendicular to length). All animal experiments performed were conducted with the approval of Institutional Animal Care and Use Committee of Peking Union Medical College Hospital.

### Statistical analysis

Statistical analyses were carried out by using GraphPad Prism 7.0 (Graphpad Software, La Jolla, CA). Data were expressed as mean ± standard deviation (SD) at least three independent experiments. Chi-square test and Spearman correlation analysis were performed to access the association between UAP1L1 expression and clinical characteristics of prostate cancer patients. One-way analysis of variance test and Student’s t-test were used for the comparison of the significant differences for multiple groups and two independent groups, respectively. It was considered to be statistically significant when *P* value was less than 0.05.

## Results

### UAP1L1 was upregulated in the human prostate cancer tissues

**Fig. 1:**
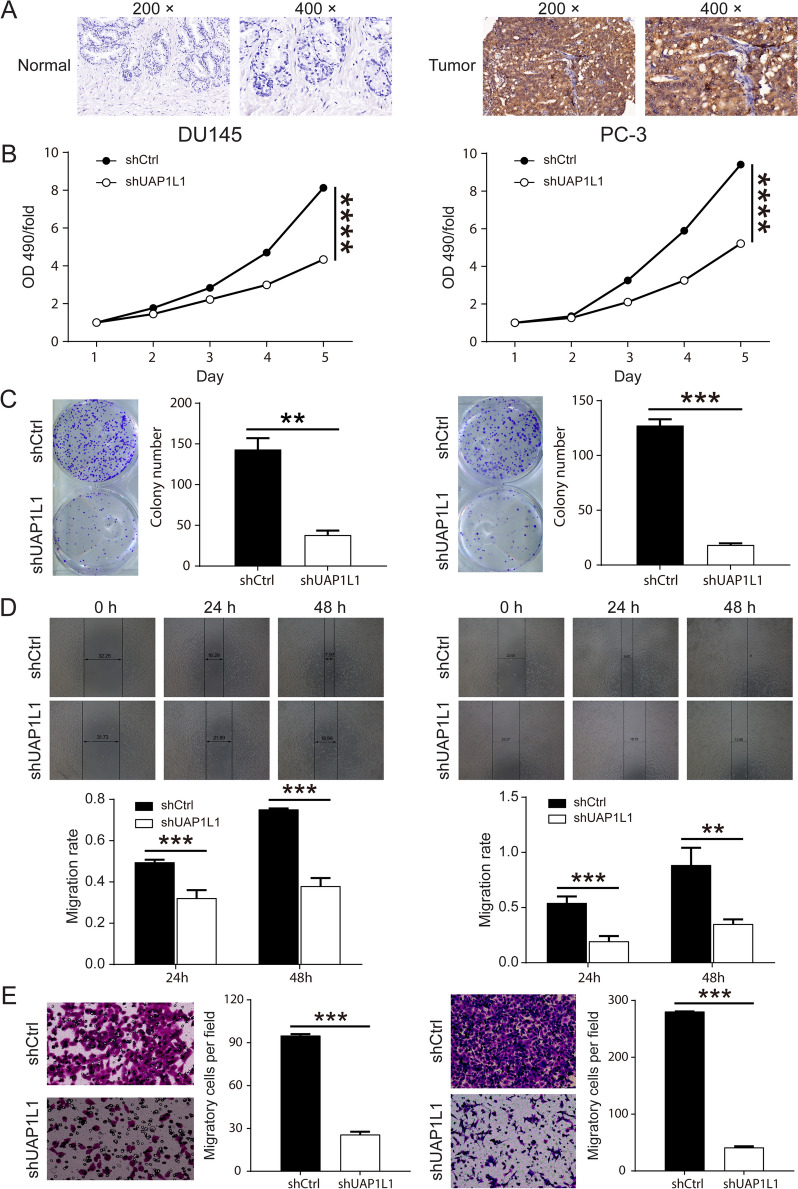
UAP1L1 upregulated in prostate cancer tissues and promoted the proliferation and migration of prostate cancer cells. **A** The expression levels of UAP1L1 was upregulated in prostate cancer tissues, which was detected by immunohischemical staining. Magnification was 200 times and 400 times. **B** MTT assay results indicated that UAP1L1 knockdown inhibited the proliferation of prostate cancer cells (DU 145 and PC-3). **C** The colony number in shUAP1L1 group was significantly decreased according to the results of clone formation assay. **D** The migration ability of prostate cancer cells was determined by wound healing assay and the migration rate was reduced after UAP1L1 knockdown. **E** The invasion ability of prostate cancer cells was detected through Transwell assay. ShCtrl: prostate cancer cells infected with shRNA of control; shUAP1L1: prostate cancer cells infected with shRNA of UAP1L1. **P < 0.01; ***P < 0.001; ****P < 0.0001

For the purpose of investigating the role of UAP1L1 in prostate cancer, the expression levels of UAP1L1 in prostate cancer tissues were firstly detected by immunohistochemical staining, which indicated that UAP1L1 was significantly upregulated in prostate cancer tissues (Fig. 1A). In addition, the expression of UAP1L1 in prostate cancer tissues was obvious higher than normal prostate tissues according to the immunohistochemistry analysis (Table 1). Correlation analysis between UAP1L1 expression and clinical characteristics in patients with prostate cancer revealed that there were significant differences in the expression of UAP1L1 in pathology grade, Gleason score and Gleason grade (Table 2). Furthermore, Spearman correlation analysis showed that the expression of UAP1L1 was positively correlated with pathology grade, Gleason score and Gleason grade, respectively (Table 3). Above all, UAP1L1 may be related to the process of prostate cancer.

**Table 1:** Expression patterns of UAP1L1 in prostate cancer tissues and normal prostate tissues revealed in immunohistochemistry analysis

UAP1L1 expression	Tumor tissue		Normal prostate tissue		*P* value
	Cases	Percentage	Cases	Percentage	
Low	81	51.3	80	100%	< 0.001***
High	77	48.7	0	–	

Note: ***P < 0.001

**Table 2:** Relationship between UAP1L1 expression and tumor characteristics in patients with prostate cancer

Features	No. of cases	UAP1L1 expression		*P* value
		Low	High	
All cases	158	81	77	
Age (years)				0.735
≤ 69	84	42	42	
> 69	74	39	35	
Gleason Score				0.012*
< 8	60	37	23	
≥ 8	91	37	54	
Grade				0.001**
1	12	12	0	
2	38	21	17	
3	101	41	60	
T Infiltrate				0.476
T1	2	2	0	
T2	68	41	27	
T3	38	22	16	
T4	6	3	3	
Lymphatic metastasis (N)				0.361
N0	106	62	44	
N1	8	6	2	
Stage				0.186
I	14	14	0	
II	56	29	27	
III	32	16	16	
IV	12	9	3	
Gleason Grade				0.022*
2	8	8	0	
3	38	21	17	
4	54	24	30	
5	50	20	30	
6	1	1	0	

Note: *P < 0.05, **P < 0.01

**Table 3:** Relationship between UAP1L1 expression and tumor characteristics in patients with prostate cancer

Tumor characteristics	Index	UAP1L1
Grade	Spearman correlation	0.273
	Significance (two-tailed)	0.001**
	N	151
Gleason score	Spearman correlation	0.206
	Significance (two-tailed)	0.011*
	N	151
Gleason grade	Spearman correlation	0.187
	Significance (two-tailed)	0.021*
	N	151

Note: *P < 0.05, **P < 0.01

### UAP1L1 promoted human prostate cancer cell growth and inhibited cell apoptosis in vitro

To explore the role of UAP1L1 in tumor growth, we used lentivirus targeting against UAP1L1 mRNA to suppress the expression of UAP1L1 in prostate cancer cell lines (DU 145 and PC3). The results showed that the lentivirus was efficiently transfected into DU 145 and PC3 cells (Additional file 1: Fig. S1A), and the qPCR analysis and WB assay identified that the expression of UAP1L1 was significantly inhibited in both cancer cells (Additional file 1: Fig. S1B, C). Subsequently, growth curve analysis showed that knockdown of UAP1L1 expression by lentivirus attenuated the growth of both prostate cancer cells (Fig. 1B). Moreover, the capacity of cell proliferation was notably suppressed through the knockdown of UAP1L1 in both DU 145 and PC3 cells according to the results of colony formulation assay (Fig. 1C).

**Fig. 2:**
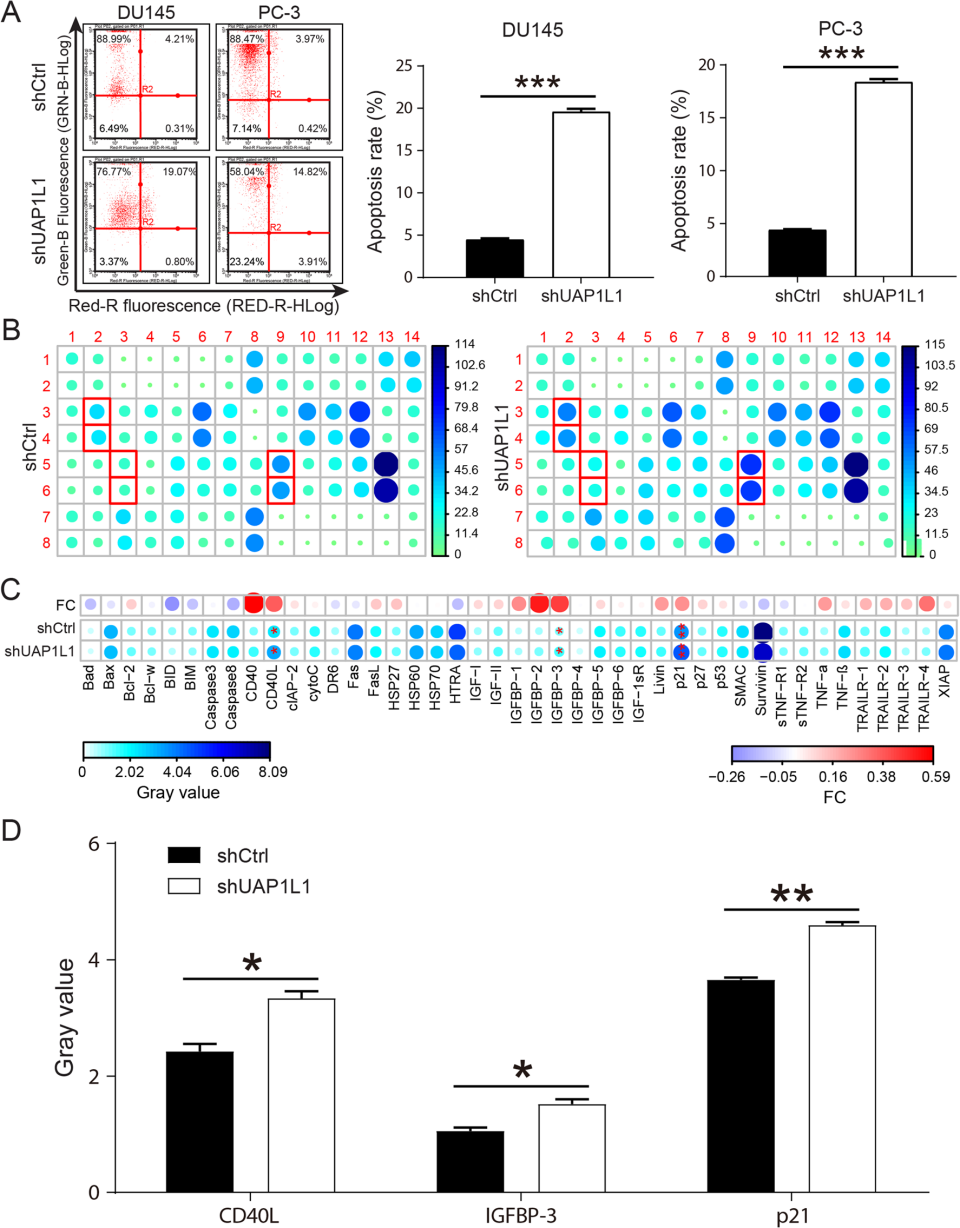
UAP1L1 knockdown inhibited the prostate cancer cells apoptosis in vitro. **A** Flow cytometry was used to detect the cell apoptosis, and the results indicated that shUAP1L1 inducted the apoptosis of prostate cancer cells. **B** The intracellular signaling array after shUAP1L1 infection was determined. The red box indicated upregulation of proteins expression. **C** The expression of proteins associated with cell apoptosis pathway in shUAP1L1 group was compared to that in shCtrl group, and the fold change was also presented. **D** The proteins with signaling changes in expression levels were shown in histograms. ShCtrl: prostate cancer cells infected with shRNA of control; shUAP1L1: prostate cancer cells infected with shRNA of UAP1L1. *P < 0.05; **P < 0.01

Next, we further examined the impact of UAP1L1 knockdown on the capacity of invasion and migration, and cell apoptosis. The capacity of migration and invasion as characteristics of malignant cells was significantly suppressed by UAP1L1 knockdown in both DU 145 and PC3 cells (Fig. 1D and E). The results showed that the knockdown of UAP1L1 remarkably enhanced the apoptosis in both prostate cancer cells (Fig. 2A). Meanwhile, the analysis of Human Apoptosis Antibody Array indicated that after UAP1L1 knockdown in PC-3 cells, the expression levels of CD40L, IGFBP-3 and p21 was obviously upregulated (Fig. 2B–D). Altogether, these results indicated that UAP1L1 knockdown inhibited prostate cancer cell proliferation, invasion and migration in vitro, but induced cell apoptosis probably through regulating the expression of CD40L, IGFBP-3 and p21.

### UAP1L1 target genes from several cancer-related pathways

**Fig. 3:**
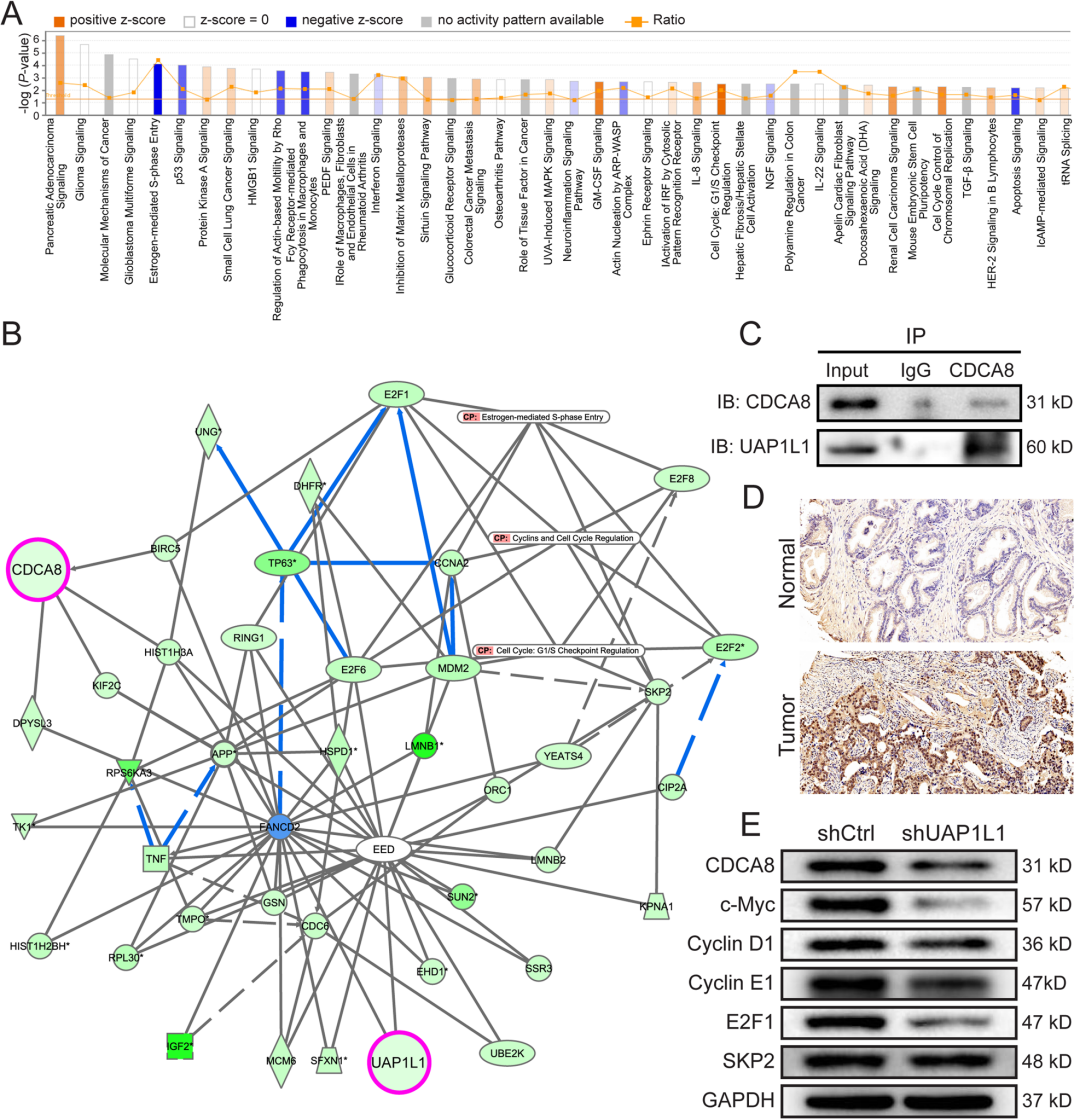
The downstream gene of UAP1L1 was screened by IPA. **A** After UAP1L1 knockdown, the state of signaling pathways in prostate cancer cells was analyzed by IPA. **B** The interaction network of genes and pathways was assessed by IPA. **C** Co-IP was used to verify the interaction between UAP1L1 and CDCA8, which was indicated that UAP1L1 protein interacted with CDCA8 protein. **D** The expression levels of CDCA8 was upregulated in prostate cancer tissues, which was detected by immunohischemical staining. Magnification was 200 times. **E** The expression of CDCA8, downstream molecules of UAP1L1, and proteins related to pathways was detected by western blot. ShCtrl: prostate cancer cells infected with shRNA of control; shUAP1L1: prostate cancer cells infected with shRNA of UAP1L1. Input: whole cell lysate of C4-2 cell group; IgG: control group by immunoprecipitation with IgG; CDCA8: protein obtained by immunoprecipitation with CDCA8 antibody.

The differentially expressed genes after UAP1L1 knockdown were analyzed by ingenuity pathway analysis (IPA) software, to elucidate which pathways and functions were mainly affected by aberrant mRNA expression. Some canonical pathways were demonstrated to be significantly inhibited, including the cell cycle: estrogen-mediated S-phase entry, p53 signaling pathway, Fcy receptor-mediated phagocytosis in macrophages and monocytes pathway, and Apoptosis signaling pathway (Fig. 3A). And the major functions of differentially expressed genes were shown in Additional file 1: Fig. S2A, which includes Cell death and survival, Cellular movement, Cellular growth and proliferation, Cell cycle and so on. What’s more, the molecular-pathway network revealed the interactions between genes involved in estrogen-mediated S-phase entry pathway, Cyclins and cell cycle regulation pathway and Cell cycle: GA/S checkpoint regulation pathway (Fig. 3B). 20 genes targeted UAP1L1 were chosen and detected the expression levels after UAP1L1 knockdown. The results suggested that 10 genes, including CDCA8, were significantly downregulated (Additional file 1: Fig. S2B). Co-IP results further confirmed the interaction between UAP1L1 protein and CDCA8 protein (Fig. 3C). The immunohistochemical staining results indicated CDCA8 had higher expression level in prostate cancer tissue than normal prostatic tissues (Fig. 3D). In addition, the protein levels of CDCA8 as well as the pathway related proteins (c-Myc, Cyclin D1, Cyclin E1 and E2F1) were also downregulated by shUAP1L1 (Fig. 3E). Therefore, CDCA8, as a downstream gene of UAP1L1 was chosen for following studies.

### UAP1L1 promoted prostate cancer by regulating CDCA8 expression in vitro

**Fig. 4:**
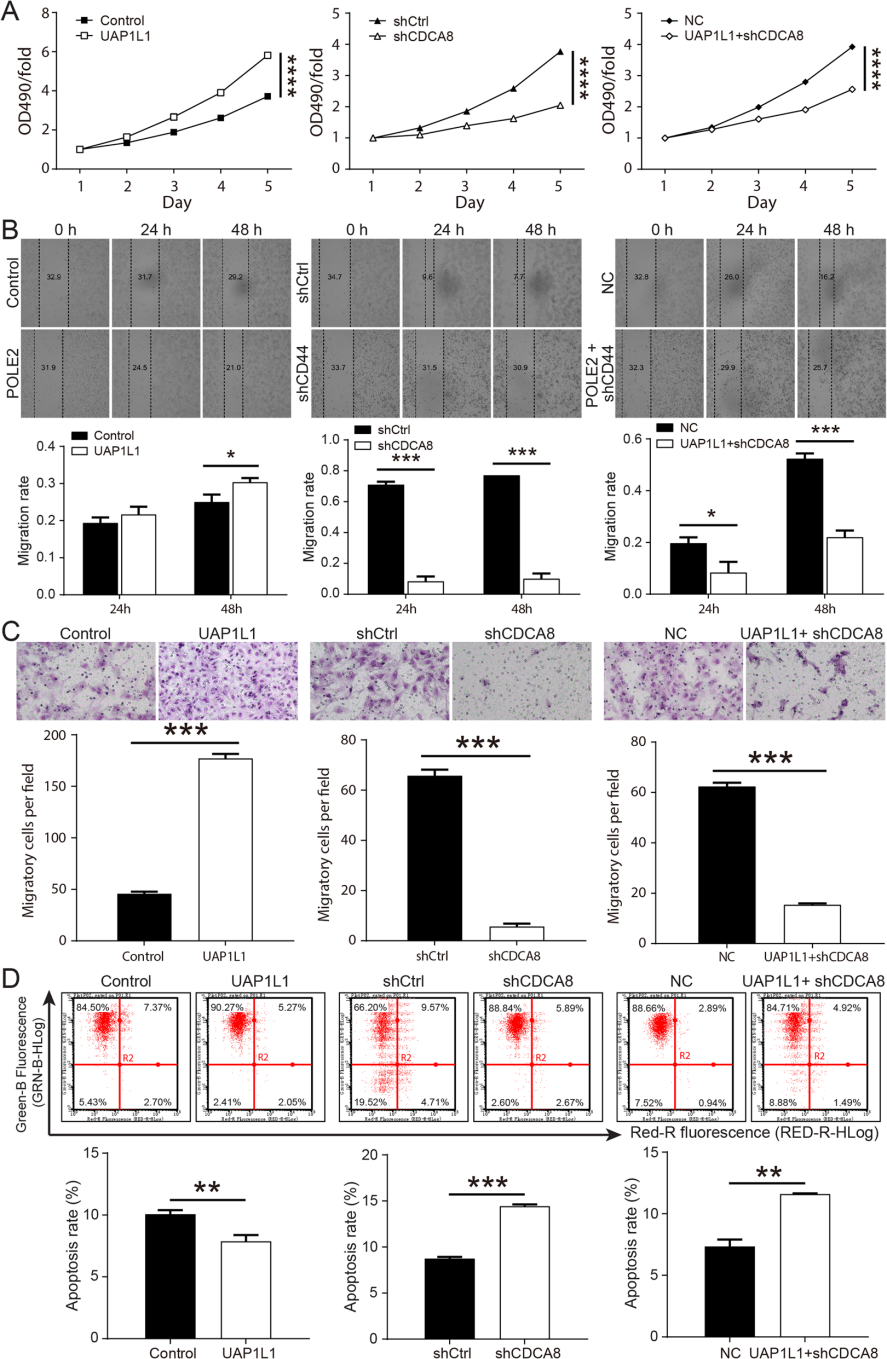
The effects of UAP1L1 overexpression and CDCA8 knockdown on prostate cancer cells were demonstrated in vitro. **A** MTT assay results indicated the influence of UAP1L1 overexpression and CDCA8 knockdown on the proliferation of prostate cancer cells. **B** The migration ability of C4-2 cells was determined by wound healing assay and the migration rate was increased by upregulating UAP1L1, but reduced after CDCA8 knockdown. **C** Transwell assay revealed that UAP1L1 overexpression promoted the invasion ability of prostate cancer cells, which could be inhibited by CDCA8 knockdown. **D** Flow cytometry was performed for detecting the apoptosis of C4-2 cells. Control, shCtrl and NC: negative control; UAP1L1: prostate cancer cells overexpressed UAP1L1; shCDCA8: prostate cancer cells infected with shRNA of CDCA8; UAP1L1 + shCDCA8: prostate cancer cells overexpressed UAP1L1 as well as infected with shRNA of CDCA8. *P < 0.05; ***P < 0.001

The UAP1L1 overexpression, CDCA8 knockdown and UAP1L1 overexpression accompanied by CDCA8 knockdown cell models were constructed successfully in vitro by lentivirus transfection in order to investigate the effects of CDCA8 regulated by UAP1L1 on prostate cancer cells (Additional file 1: Fig. S3A). We found that the mRNA and proteins levels of UAP1L1 were significantly upregulated in UAP1L1 overexpression group, and the levels of CDCA8 were markedly downregulated in CDCA8 knockdown group. However, this effect of promoting or inhibiting expression was alleviated in the UAP1L1 overexpression accompanied by CDCA8 knockdown group (Additional file 1: Fig. S3B, C). According to the results of MTT, UAP1L1 overexpression promoted C4-2 cells proliferation, whereas CDCA8 knockdown suppressed (Fig. 4A). In addition, the migration and invasion of C4-2 cells were promoted by UAP1L1 and inhibited by shCDCA8. But the inhibitory effect of shCDCA8 would be weakened, when prostate cancer cells overexpressed UAP1L1 as well as knocked down CDCA8 (Fig. 4B, C). UAP1L1 reduced the levels of cell apoptosis. On the contrary, shCDCA8 significantly promoted cell apoptosis because the apoptosis rate increased by 1.52 times, and the upregulation of CDCA8 reduced the increase of apoptosis rate (Fig. 4D).

### UAP1L1 promoted the growth of prostate cancer tumor in vivo

**Fig. 5:**
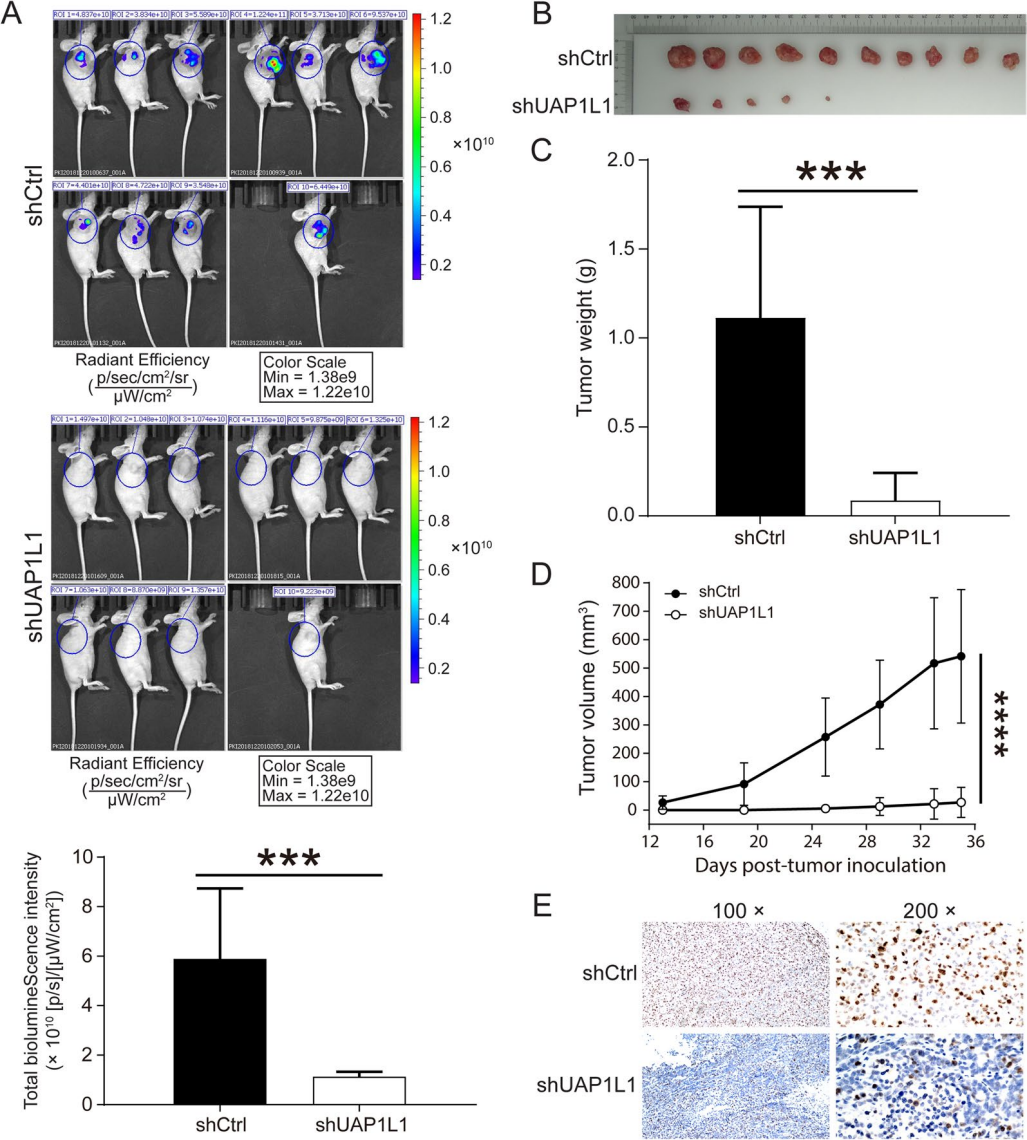
The effects of UAP1L1 on prostate cancer were verified in vivo. **A** The images of mice by bioluminescence imaging were provided and the bioluminescence intensity was tested. **B** The solid tumors were removed and photographed after the mice were sacrificed. **C** The tumors from mice were weighted. **D** The volume of tumors in mice was measured at designated point in time with Vernier caliper. **E** The Ki67 expression in tumor tissue from mice was determined by immunohischemical staining. Magnification was 100 times and 200 times. ShCtrl: prostate cancer cells infected with shRNA of control; shUAP1L1: prostate cancer cells infected with shRNA of UAP1L1. ***P < 0.001; ****P < 0.0001

After successfully constructing UAP1L1 knockdown mouse model, the results of in vivo bioluminescence imaging showed that the total bioluminescence intensity was markedly decreased in shUAP1L1 group (Fig. 5A). The representative images of solid tumor obtained from mice clearly showed a reduced speed of tumor growth after UAP1L1 downregulation (Fig. 5B). Moreover, the smaller volume and lighter weight of soil tumors in the shUAP1L1 group also suggested that tumor growth slowed down (Fig. 5C, D). Besides, the immunohistochemical staining results of tumor tissues from mice in Fig. 5E revealed that the expression of Ki67 was significantly downregulated, suggesting that the proliferation of prostate cancer cells in vivo was inhibited by UAP1L1 knockdown. These results indicated that downregulation of UAP1L1 suppressed tumor growth in vivo.

## Discussion

Prostate cancer was the second most common cancer in men, causing more than 300,000 deaths each year all of the world [12]. The incidence of prostate cancer increased steadily with age [13], especially in men over 79 years old [14]. The current mainstay treatment for prostate cancer was androgen-deprivation therapies (ADT) [15], and it was effective initially but the prognoses of advanced-prostate cancer patients remained poor on account of the cancer metastasis [16]. Therefore, it was also necessary to screen novel potential target genes for predicting the risk of prostate cancer progression and the treatment.

In the present study, we found UAP1L1 was upregulated in human prostate cancer tissues, and the expression of UAP1L1 was positively correlated with pathology grade, Gleason score and Gleason grade of patients with prostate cancer. According to the study of Weiqiang Li et al*.*, some clinical characteristics, including age at diagnosis, Gleason score, PSA levels and cancer stage, were established predictors for the risk of prostate cancer progression [17]. Hence, UAP1L1 might serve as a potential target to predict the risk of prostate cancer progression. Although there was few researches that studied the role of UAP1L1 in prostate cancer, Ching-Yu Lai et al*.* demonstrated that UAP1L1 was significantly upregulated in hepatocellular carcinoma tissues and UAP1L1 knockdown attenuated the growth of hepatocellular carcinoma cells [11]. Furthermore, studies determined that UAP1L1 knockdown inhibited the proliferation of glioma and esophageal squamous cell carcinoma cells and tumor growth in vivo [18, 19]. Likewise, the results of this study demonstrated that UAP1L1 knockdown inhibited proliferation, clone formation ability, and migration of prostate cancer cells (DU 145 and PC-3), and promoted the cell apoptosis. The apoptosis-related proteins (CD40L, IGFBP-3 and p21) were upregulated in the shUAP1L1 group in the Human Apoptosis Antibody Array analysis, suggesting that UAP1L1 knockdown might induced cell apoptosis via regulating the expression of CD40L, IGFBP-3 and p21.

Previous studies showed that UAP1L1 acted as a tumor promoter in the progression of gastric cancer by regulating the cell cycle regulator CDK6 [20]. However, the mechanism of action of UAP1L1 in prostate cancer was still unclear. In this study, through the screening of differentially expressed genes and the preliminary analysis of IPA, tit was found that most of downregulated genes after UAP1L1 knockdown were proved to be enriched in Estrogen-mediated S-phase Entry pathway, Cyclins and cell cycle regulation pathway and Cell cycle: G1/S checkpoint regulation pathway. Furthermore, the expression of CDCA8 was upregulated in prostate cancer tissues and inhibited by UAP1L1 knockdown. Co-IP assay revealed that CDCA8 could directly interact with UAP1L1 and was therefore chosen for subsequent studies. CDCA8, alternatively known as Borealin, was one of the cell cycle division-associated (CDCA) protein family [21], and part of the chromosomal passenger complex [22]. As a pivotal regulatory factor of mitosis and cell division, CDCA8 functioned to properly align and segregate chromosomes during mitosis [22, 23]. CDCA8 was considered to be a hypothetical oncogene, upregulated in many types of cancer tissues, but very low or absent in normal tissues [24], which was consistent with our findings. Besides, the study of CHAO CI et al*.* indicated that CDCA8 knockdown inhibited cell proliferation and migration of cutaneous melanoma cells [25]. Zhengshuai Song et al*.* revealed that CDCA8 might promote the tumorigenesis and progression of prostate cancer, and that the changes of cell cycle and p53 signaling pathway were two major signatures of prostate cancer by using bioinformatics analysis [26]. However, the specific role of CDCA8 in prostate cancer was not confirmed in experiments. In the present study, the results of cell function experiments suggested that the downregulation of CDCA8 inhibited the proliferation and migration of prostate cancer cells and promoted apoptosis. UAP1L1 knockdown reduced CDCA8 expression in prostate cancer cells. Besides, the overexpression of UAP1L1 promoted the proliferation and migration of prostate cancer cells, which also alleviated the inhibitory effects of CDCA8 knockdown on prostate cancer progression. UAP1L1 knockdown also inhibited the proliferation of prostate cancer cells in vivo and further suppressed the growth of tumor in mice models.

## Conclusions

To sum up the foregoing, UAP1L1 knockdown inhibited the prostate cancer cells proliferation and migration in vitro, and tumor growth in vivo. Besides, the regulation mechanism of UAP1L1 in prostate cancer was explored preliminarily, which revealed that UAP1L1 promoted prostate cancer progression by regulating CDCA8. Therefore, UAP1L1/CDCA8 can be used as potential therapeutic target for prostate cancer, providing a new possible strategy for the diagnosis and treatment of prostate cancer. However, the exact mechanisms and in-depth regulation mode between UAP1L1 and CDCA8 should be explored in the future.

## Supplementary Information

**Additional file 1: Figure S1.** The UAP1L1 knockdown cell model was constructed in vitro. **Figure S2.** The results of diseases and function enrichment and the filtrate of downstream molecules of UAP1L1. **Figure S3.** The UAP1L1 overexpression, CDCA8 knockdown, and UAP1L1 overexpression as well as CDCA8 knockdown cell models were constructed in vitro*.* **Table S1.** The information of antibodies used in western blot and immunohistochemical staining (IHC). **Table S2.** Target sequences and shRNA sequences used for gene knockdown. **Table S3.** The primer sequences of genes used for qRT-PCR

## Data Availability

The datasets used and/or analysed during the current study are available from the corresponding author on reasonable request.

## References

[1] Stowell SR, Ju T, Cummings RD (2015). Protein glycosylation in cancer. Annu Rev Pathol.

[2] Wagner KW (2007). Death-receptor O-glycosylation controls tumor-cell sensitivity to the proapoptotic ligand Apo2L/TRAIL. Nat Med.

[3] Ashkenazi A (1999). Safety and antitumor activity of recombinant soluble Apo2 ligand. J Clin Invest.

[4] Ju T, Otto VI, Cummings RD (2011). The Tn antigen-structural simplicity and biological complexity. Angew Chem Int Ed Engl.

[5] Ju T (2013). Tn and sialyl-Tn antigens, aberrant O-glycomics as human disease markers. Proteomics Clin Appl.

[6] Butkinaree C, Park K, Hart GW (2010). O-linked beta-N-acetylglucosamine (O-GlcNAc): Extensive crosstalk with phosphorylation to regulate signaling and transcription in response to nutrients and stress. Biochim Biophys Acta.

[7] Capotosti F (2011). O-GlcNAc transferase catalyzes site-specific proteolysis of HCF-1. Cell.

[8] Haltiwanger RS, Holt GD, Hart GW (1990). Enzymatic addition of O-GlcNAc to nuclear and cytoplasmic proteins. Identification of a uridine diphospho-*N*-acetylglucosamine:peptide beta-N-acetylglucosaminyltransferase. J Biol Chem.

[9] Peneff C (2001). Crystal structures of two human pyrophosphorylase isoforms in complexes with UDPGlc(Gal)NAc: role of the alternatively spliced insert in the enzyme oligomeric assembly and active site architecture. EMBO J.

[10] Hill VK (2011). Genome-wide DNA methylation profiling of CpG islands in breast cancer identifies novel genes associated with tumorigenicity. Cancer Res.

[11] Lai CY (2019). Identification of UAP1L1 as a critical factor for protein O-GlcNAcylation and cell proliferation in human hepatoma cells. Oncogene.

[12] Bray F (2018). Global cancer statistics 2018: GLOBOCAN estimates of incidence and mortality worldwide for 36 cancers in 185 countries. Cancer J Clin.

[13] Russo JW, Balk SP (2018). Initiation and evolution of early onset prostate cancer. Cancer Cell.

[14] Farashi S, Kryza T, Clements J, Batra J (2019). Post-GWAS in prostate cancer: from genetic association to biological contribution. Nat Rev Cancer.

[15] Zhang Y (2018). Androgen deprivation promotes neuroendocrine differentiation and angiogenesis through CREB-EZH2-TSP1 pathway in prostate cancers. Nat Commun.

[16] Shin SH (2018). Aberrant expression of CITED2 promotes prostate cancer metastasis by activating the nucleolin-AKT pathway. Nat Commun.

[17] Li W (2018). Genome-wide scan identifies role for AOX1 in prostate cancer survival. Eur Urol.

[18] Yang Z (2021). UAP1L1 plays an oncogene-like role in glioma through promoting proliferation and inhibiting apoptosis. Ann Transl Med.

[19] Xiao X (2021). Silencing of UAP1L1 inhibits proliferation and induces apoptosis in esophageal squamous cell carcinoma. Mol Carcinog.

[20] Qi J, Liu S, Liu W, Cai G, Liao G (2020). Identification of UAP1L1 as tumor promotor in gastric cancer through regulation of CDK6. Aging (Albany NY).

[21] Phan NN (2018). Distinct expression of CDCA3, CDCA5, and CDCA8 leads to shorter relapse free survival in breast cancer patient. Oncotarget.

[22] Lawrenson K (2015). Cis-eQTL analysis and functional validation of candidate susceptibility genes for high-grade serous ovarian cancer. Nat Commun.

[23] Zhao S (2019). Molecular portraits and trastuzumab responsiveness of estrogen receptor-positive, progesterone receptor-positive, and HER2-positive breast cancer. Theranostics.

[24] Dai C (2015). Transcriptional activation of human CDCA8 gene regulated by transcription factor NF-Y in embryonic stem cells and cancer cells. J Biol Chem.

[25] Ci C (2019). Overexpression of CDCA8 promotes the malignant progression of cutaneous melanoma and leads to poor prognosis. Int J Mol Med.

[26] Song Z (2019). The identification of potential biomarkers and biological pathways in prostate cancer. J Cancer.

