# Supplementary material for: Identification of UAP1L1 as a critical factor for prostate cancer and underlying molecular mechanism in tumorigenicity
Source: J Transl Med. 2022 Feb 15;20:91. doi: 10.1186/s12967-022-03291-0 (PMC8845250; doi:10.1186/s12967-022-03291-0)
Supplement: Supplementary file 1 — Additional file 1: Figure S1. The UAP1L1 knockdown cell model was constructed in vitro. Figure S2. The results of diseases and function enrichment and the filtrate of downstream molecules of UAP1L1. Figure S3. The UAP1L1 overexpression, CDCA8 knockdown, and UAP1L1 overexpression as well as CDCA8 knockdown cell models were constructed in vitro. Table S1. The information of antibodies used in western blot and immunohistochemical staining (IHC). Table S2. Target sequences and shRNA sequences used for gene knockdown. Table S3. The primer sequences of genes used for qRT-PCR [file 12967_2022_3291_MOESM1_ESM.docx]

**
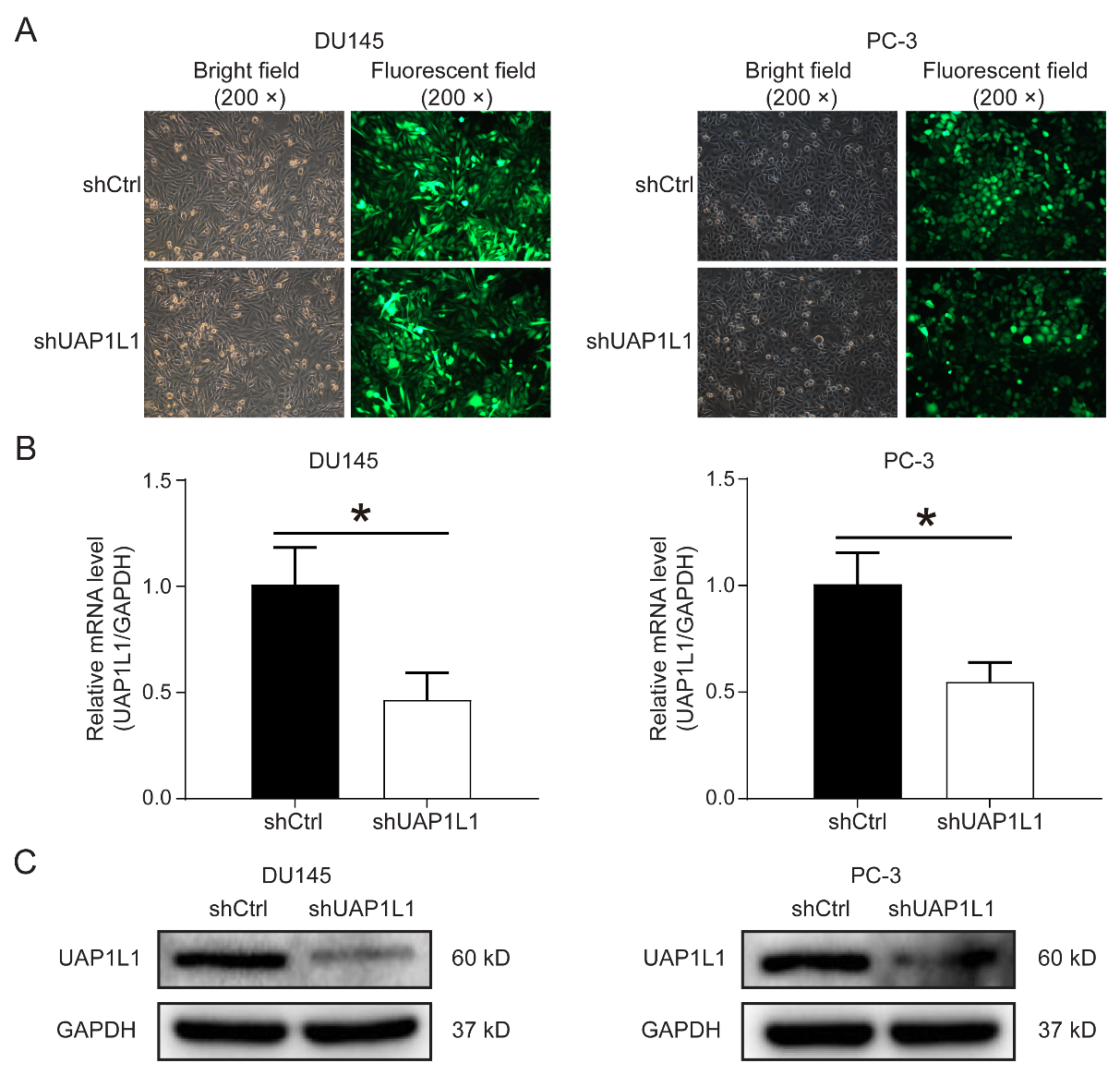
**

**Figure S1 The UAP1L1 knockdown cell model was constructed *in vitro***

(A) The infection efficiency of lentivirus with shRNA was demonstrated by observing the fluorescence expression of green fluorescence protein. Magnification was and 200 times. (B) The mRNA levels of UAP1L1 were detected by qRT-PCR after UAP1L1 knockdown. (C) The protein levels of UAP1L1 were detected by western blot after UAP1L1 knockdown. ShCtrl: prostate cancer cells infected with shRNA of control; shUAP1L1: prostate cancer cells infected with shRNA of UAP1L1. * *P* < 0.05.

**
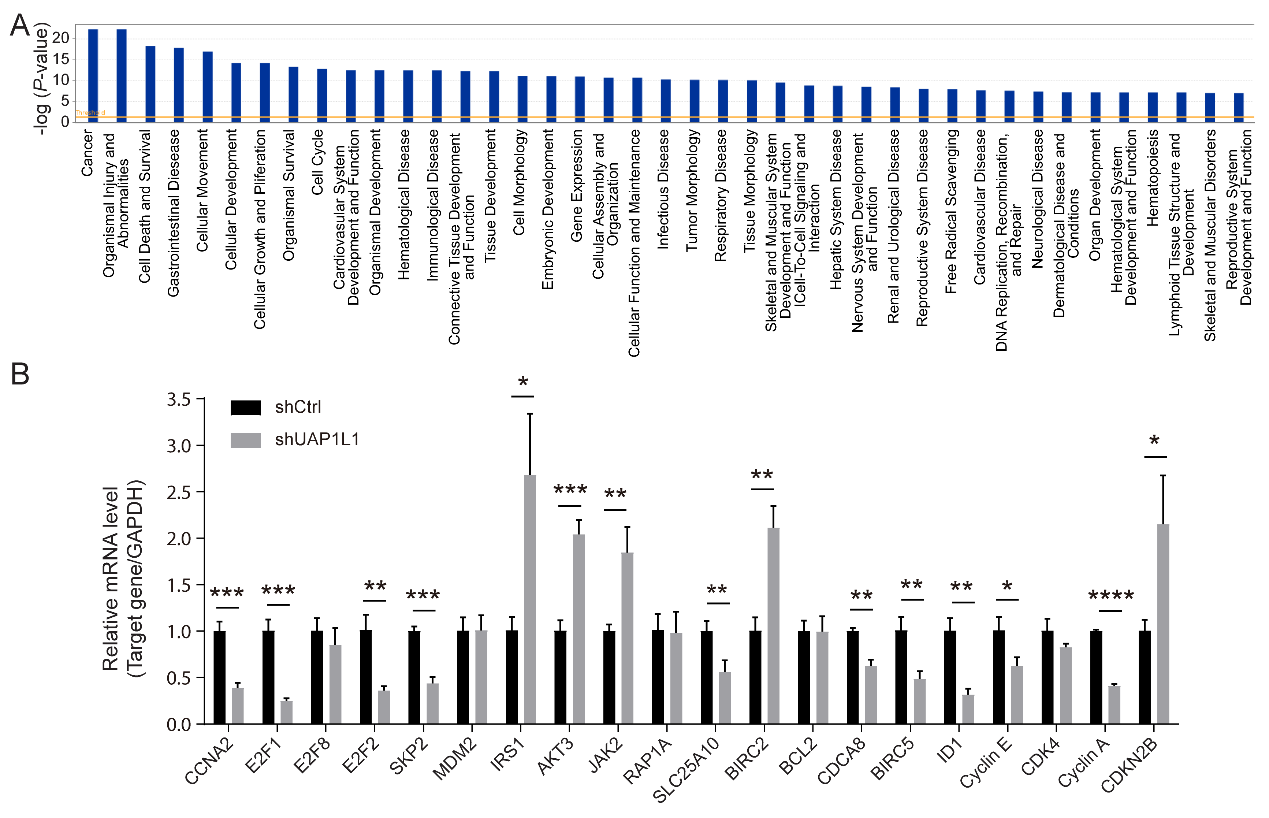
**

**Figure S2 The results of diseases and function enrichment and the filtrate of downstream molecules of UAP1L1**

(A) Diseases and function enrichment of differentially expressed genes after UAP1L1 knockdown were shown using IPA. (B) The expression of 20 downstream genes of UAP1L1 was detected to screen one of them for the following study. ShCtrl: prostate cancer cells infected with shRNA of control; shUAP1L1: prostate cancer cells infected with shRNA of UAP1L1. * *P* < 0.05; ** *P* < 0.01; *** *P* < 0.001; **** *P* < 0.0001.

**
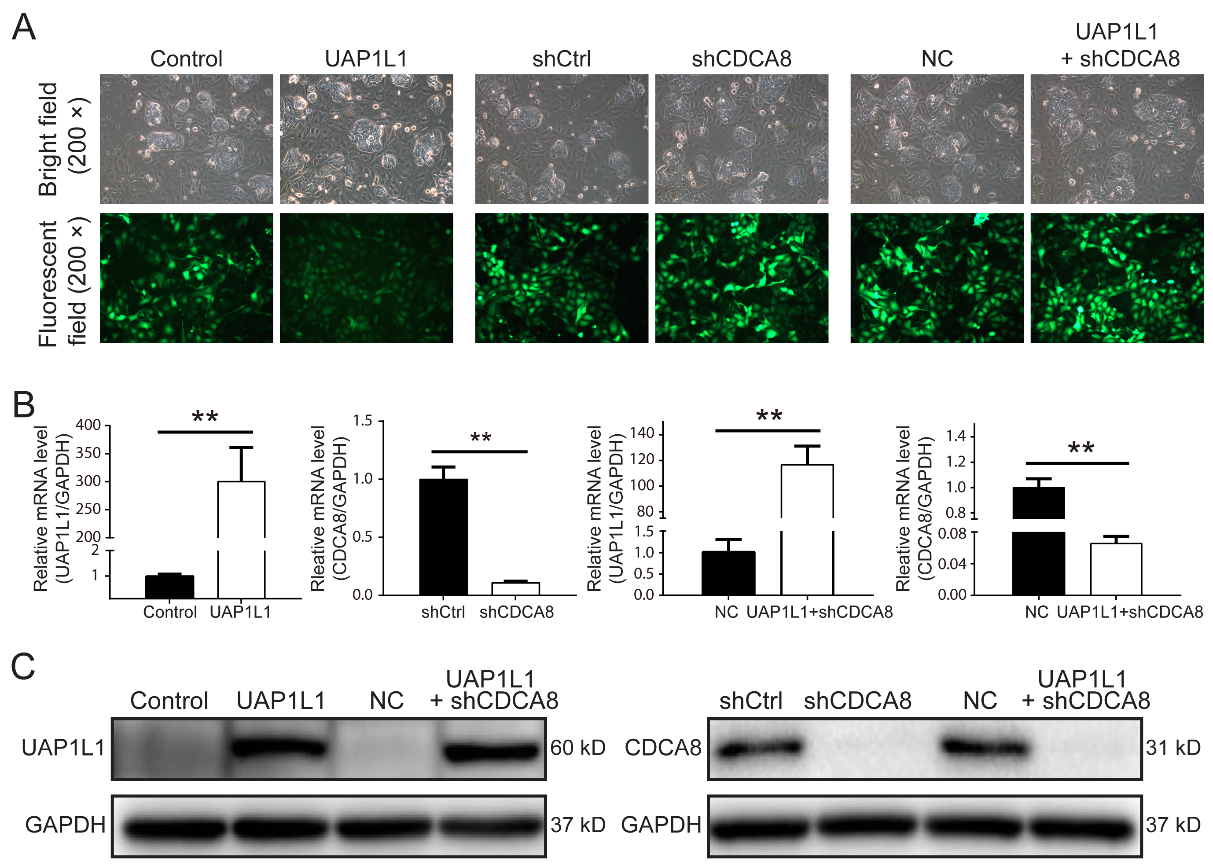
**

**Figure S3 The UAP1L1 overexpression, CDCA8 knockdown, and UAP1L1 overexpression as well as CDCA8 knockdown cell models were constructed *in vitro***

(A) The infection efficiency of lentivirus was demonstrated by observing the fluorescence expression of green fluorescence protein. Magnification was and 200 times. (B) The mRNA levels of UAP1L1 and CDCA8 were detected by qT-PCR. (C) The protein levels of UAP1L1 and CDCA8 were inspected by western blot. Control, shCtrl and NC: negative control; UAP1L1: prostate cancer cells overexpressed UAP1L1; shCDCA8: prostate cancer cells infected with shRNA of CDCA8; UAP1L1 + shCDCA8: prostate cancer cells overexpressed UAP1L1 as well as infected with shRNA of CDCA8. ** *P* < 0.01.

**Table S1.** The information of antibodies used in western blotting and IHC

| Primary antibodies | Dilution | Source species | Company | Catalog No. |
| --- | --- | --- | --- | --- |
| UAP1L1 (WB) | 1:1000 | Rabbit | abcam | ab174855 |
| CDCA8 (WB) | 1:1000 | Rabbit | abcam | ab70910 |
| c-Myc (WB) | 1:1000 | Rabbit | CST | 5605 |
| Cyclin D1 (WB) | 1:1000 | Rabbit | CST | 2978 |
| Cyclin E1 (WB) | 1:2000 | Mouse | abcam | ab3927 |
| E2F1 (WB) | 1:1000 | Rabbit | abcam | ab179445 |
| SKP2 (WB) | 1:500 | Rabbit | abcam | ab183039 |
| GAPDH (WB) | 1:3000 | Rabbit | Bioworld | AP0063 |
| UAP1L1 (IHC) | 1:200 | Rabbit | abcam | ab174855 |
| CDCA8 (IHC) | 1:20 | Rabbit | abcam | ab70910 |
| Ki67 (IHC) | 1:200 | Rabbit | abcam | ab16667 |
|  |  |  |  |  |
| Secondary antibody | Dilution |  | Company | Catalog No. |
| HRP Goat Anti-Rabbit IgG (WB) | 1:3000 |  | Beyotime | A0208 |
| HRP Goat Anti-Mouse IgG (WB) | 1:3000 |  | Beyotime | A0216 |
| HRP Goat Anti-Rabbit IgG (IHC) | 1:400 |  | abcam | ab6721 |

**Table S2.** Target sequences and shRNA sequences used for gene knockdown

| Gene symbol | Target sequence |  | shRNA sequences (5'-3') |
| --- | --- | --- | --- |
| UAP1L1 | GCCCTTCTTACTGCAAACCAT | Pbr10328 | ccggGCCCTTCTTACTGCAAACCATctcgagATGGTTTGCAGTAAGAAGGGCtttttg |
|  |  |  | aattcaaaaaGCCCTTCTTACTGCAAACCATctcgagATGGTTTGCAGTAAGAAGGGC |
| CDCA8 | GTGGAAATACGAATCAAGCAA | Pbr10540 | ccggAGATGAAATGATAGTGGAAGActcgagTCTTCCACTATCATTTCATCTtttttg |
|  |  |  | aattcaaaaaAGATGAAATGATAGTGGAAGActcgagTCTTCCACTATCATTTCATCT |
| Negative control | TTCTCCGAACGTGTCACGT |  | ccggTTCTCCGAACGTGTCACGTttcaagagaACGTGACACGTTCGGAGAAtttttg |
|  |  |  | aattcaaaaaTTCTCCGAACGTGTCACGTtctcttgaaACGTGACACGTTCGGAGAA |

**Table S3.** The primer sequences of genes used for RTPCR

| Gene | Forward primer sequence (5'-3') | Reverse primer sequence (5'-3') |
| --- | --- | --- |
| UAP1L1 | GGAGCGGAAAGACAAAGTTGC | CACAGAAGCCGATGAAGACAGG |
| CCNA2 | AGCCTGCGTTCACCATTCA | GGGCATCTTCACGCTCTATTTT |
| E2F1 | CACTTTCGGCCCTTTTGCTC | GTGCTCTCACCGTCCTACAC |
| E2F8 | TGACGAAGTGGCAGAGGAAC | CATCATAATCTGCTCGGCGTA |
| E2F2 | AAGGGGAAGTGCATCAGAGTG | CCAGCGAAGTGTCATACCGA |
| SKP2 | ATAGAAGTGTCCACCCTCCACG | CACCCAGAAAGGTTAAGTCGC |
| MDM2 | GGTGAGGAGCAGGCAAATGT | CGAAGCTGGAATCTGTGAGGT |
| IRS1 | GGTGGATGACTCTGTGGTGG | GGACGCTGATGGGGTTAGAG |
| AKT3 | TCCTTCCAGACAAAAGACCG | GAATGTAGATAGTCCAAGGCAGAG |
| JAK2 | CAAACCAAGAGGGTTCAAATG | GCTGGAGGTGCTACTTCTTTAC |
| RAP1A | ACGGGTTAAGGACACGGAA | TGCCAACTACTCGCTCATCTT |
| SLC25A10 | GACAGATGACCTACTCCCTGACTC | ACGAAGCCTCCAGCTAAACC |
| BIRC2 | CTTACTCCAGCCTTTCTCCAAA | GCATAACTGTAGGGGTTAGTCCTC |
| BCL2 | TGTGGCCTTCTTTGAGTTCG | ATCCCAGCCTCCGTTATCCT |
| CDCA8 | AGACAGGCAGAACCTCCTCAAG | TTCCTCCAAGGGCGAAGTAGTC |
| BIRC5 | TCTCAAGGACCACCGCATCT | TTTGCATGGGGTCGTCATCT |
| ID1 | ACGACATGAACGGCTGTTACTC | CTCCAACTGAAGGTCCCTGAT |
| Cyclin E | CTGGATGTTGACTGCCTTGAA | TGTCGCACCACTGATACCCT |
| CDK4 | CTACCAGATGGCACTTACACCC | GCAAAGATACAGCCAACACTCC |
| Cyclin A | GCCTGCGTTCACCATTCAT | CAGGGCATCTTCACGCTCTA |
| CDKN2B | GCGAGGAGAACAAGGGCA | GCACCTTCTCCACTAGTCCC |
